# Systemic Therapy for Advanced Hepatocellular Carcinoma with Impaired Liver Function: Navigating the Evidence Gap

**DOI:** 10.3390/cancers18183048

**Published:** 2026-09-20

**Authors:** Arathi Mohan, Nataliya Razumilava, Hannah Abraham, Maisha Anika, Oxana V. Crysler

**Affiliations:** 1 Division of Hematology and Oncology, Department of Internal Medicine, University of Michigan, Ann Arbor, MI 48109, USA; 2 Rogel Cancer Center, University of Michigan, Ann Arbor, MI 48109, USA; 3 Division of Gastroenterology and Hepatology, Department of Internal Medicine, University of Michigan, Ann Arbor, MI 48109, USA; 4 Department of Internal Medicine, University of Michigan, Ann Arbor, MI 48109, USA

**Keywords:** hepatocellular carcinoma, impaired liver function, systemic therapy, Child–Turcotte–Pugh, ALBI, immunotherapy, tyrosine kinase inhibitors

## Abstract

**Background**: Hepatocellular carcinoma (HCC) arises in the context of underlying cirrhosis in approximately 85% of cases, making hepatic reserve a major determinant of systemic treatment tolerance and clinical outcomes. Despite this, patients with impaired liver function but a life expectancy sufficient to potentially derive benefit from systemic therapy remain underrepresented in pivotal phase III trials, which enroll patients with well-preserved liver function (Child–Turcotte–Pugh [CTP] A). This discrepancy has created a substantial evidence gap, with treatment decisions in clinical practice relying on retrospective reports, small prospective trials, and observational registry data. **Evidence Summary**: Current evidence suggests that immune checkpoint inhibitors (ICIs) may represent a rational therapeutic approach in selected patients with impaired hepatic function, given their minimal dependence on hepatic metabolic pathways and the available safety and efficacy data in patients with CTP-A and ALBI grade 2/3 or selected CTP-B disease. Cohort 5 of the CheckMate 040 nivolumab trial included selected patients with CTP-B7/B8 disease and demonstrated modest efficacy, with an objective response rate of 12% and a median overall survival of 7.6 months. Additionally, the phase 3b single-arm SIERRA study reported a manageable safety profile for durvalumab plus tremelimumab (STRIDE regimen) in the CTP-B population. In contrast, regimens incorporating vascular endothelial growth factor (VEGF) inhibition, such as atezolizumab–bevacizumab and tyrosine kinase inhibitors (TKIs), may require cautious use due to an increased risk of bleeding or hepatic decompensation. TKIs, including sorafenib, lenvatinib, regorafenib, and cabozantinib, are metabolized by hepatic CYP450 pathways and require careful patient selection, monitoring, and dose optimization. Among TKIs, sorafenib has the most robust observational evidence in patients with impaired liver function, although survival is reduced due to concurrent liver disease. **Conclusions**: Clinical management should prioritize assessment of hepatic reserve, including bilirubin, albumin, ascites, variceal bleeding risk, portal hypertension, and encephalopathy, alongside performance status. Systemic treatment selection should favor clinical trial enrollment when feasible or, alternatively, immunotherapy-based approaches in selected CTP-B patients, with close monitoring for decompensation. Decreased liver reserve represents a heterogeneous clinical state, and treatment benefits depend on maintaining treatment duration without precipitating hepatic decompensation. Future research must prospectively incorporate populations with impaired liver function to better define efficacy, safety, and endpoints related to hepatic deterioration in this high-risk group.

## Simple Summary

Most hepatocellular carcinomas, the most common type of liver cancer, develop in people with underlying cirrhosis. Reduced liver function can affect treatment tolerance and outcomes, yet patients with impaired liver function remain underrepresented in clinical trials. This narrative review summarizes the limited evidence on systemic treatments across different degrees of liver impairment. Available studies suggest that treatments including immunotherapy and tyrosine kinase inhibitors may be feasible for carefully selected patients with moderately impaired liver function, although their benefits and risks remain uncertain. Treatment selection requires individualized consideration of liver function, fluid accumulation, confusion caused by liver disease, increased pressure within the liver’s blood vessels, bleeding risk, and overall physical condition. By summarizing current evidence and remaining uncertainties, this review may support clinical decision-making and help researchers design prospective studies that better represent patients with impaired liver function.

## 1. Introduction

**Figure 1:**
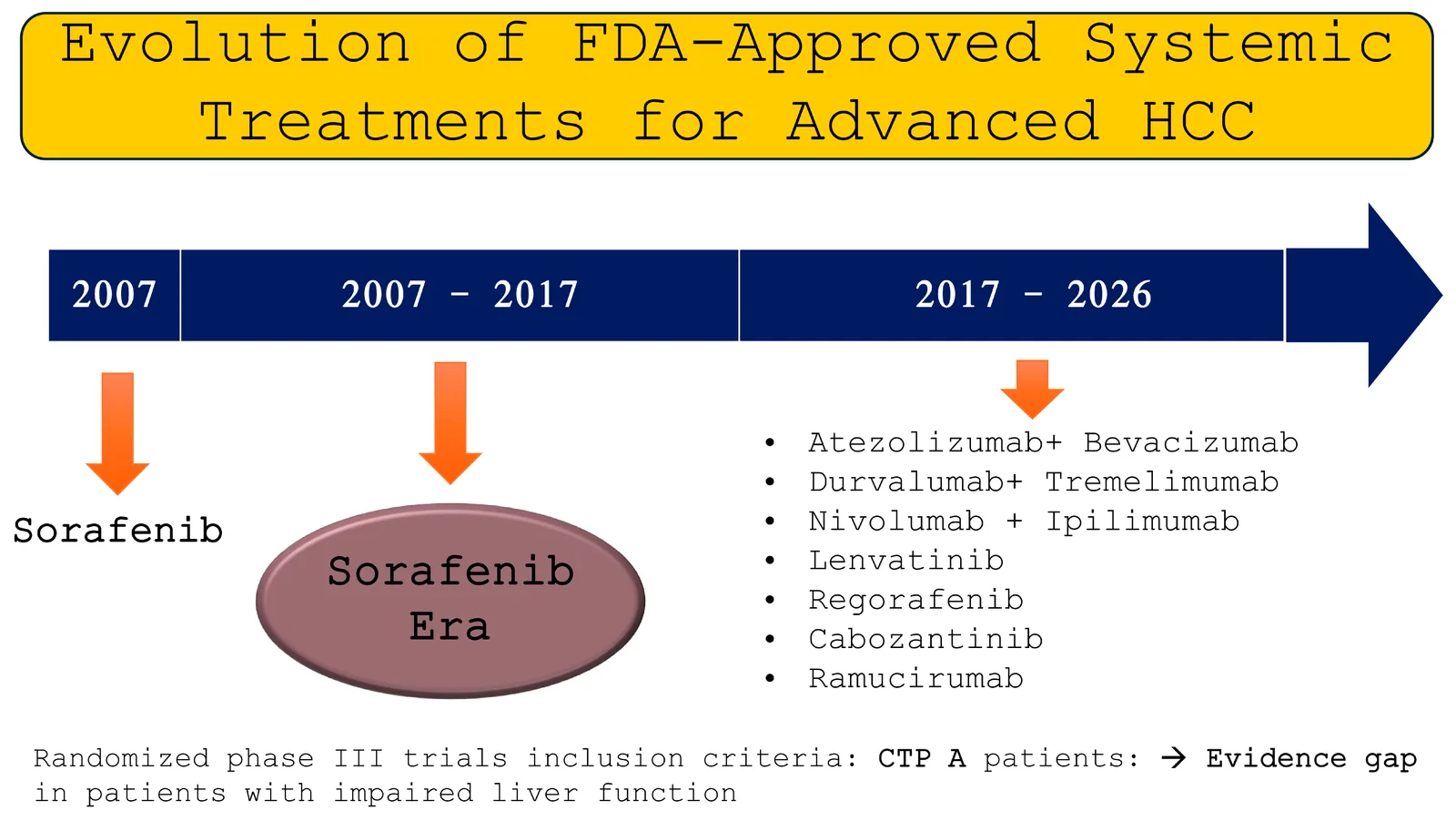
Evolution of FDA-approved systemic treatments for advanced hepatocellular carcinoma (HCC).

Hepatocellular carcinoma (HCC) is the dominant histological subtype of primary liver cancer, accounting for 75–85% of all primary liver cancers, and is the third leading cause of cancer-related death worldwide [1]. Systemic therapy for HCC has advanced substantially over the past two decades. Following the first approval of sorafenib in 2007, systemic treatment entered a prolonged sorafenib era, with rapid therapeutic expansion beginning in 2017 with the approval of regorafenib and the introduction of immune checkpoint inhibitors and additional tyrosine kinase inhibitors (Figure 1) [2,3]. However, a substantial randomized evidence gap remains, as eligibility in pivotal phase III trials of systemic therapies has been restricted to patients with well-preserved liver function, Child–Turcotte–Pugh (CTP)-A, while patients with impaired hepatic function remain underrepresented in prospective trials [2,3,4,5,6,7]. An additional limitation is that impaired liver function is not uniformly defined across studies [4,5].

The pivotal trials do not fully reflect real-world clinical practice. As HCC arises in the setting of underlying cirrhosis in approximately 85% of patients, hepatic reserve becomes a major determinant of treatment tolerance and clinical outcomes [1,8]. Consequently, treatment decisions for patients with more impaired, yet compensated or controlled, liver function often rely on retrospective studies, small prospective cohorts, post hoc analyses, and observational registry data [4,5,9]. In this population, the competing risks of tumor progression, hepatic decompensation, and treatment-related toxicity are particularly important, as limited hepatic reserve may affect both treatment tolerability and the ability to achieve sustained antitumor benefit [4,5].

The purpose of this review is to discuss practical considerations for systemic therapy across the spectrum of impaired hepatic function in patients with HCC. We evaluate the available evidence using both CTP and albumin–bilirubin (ALBI) scoring systems, including data from patients with CTP-B disease, the limited evidence available for patients with CTP-C disease, ALBI grade 2/3 analyses, and real-world clinical assessment. We aim to integrate these data to balance antitumor efficacy against the risks of hepatic decompensation and treatment toxicity when considering systemic treatment for advanced HCC and to identify priorities for future research.

This review focuses on systemic therapy for advanced hepatocellular carcinoma and does not address locoregional–systemic treatment strategies. Relevant English-language publications were identified through searches of PubMed/MEDLINE through August 1, 2026, using terms related to hepatocellular carcinoma, impaired liver function, Child–Turcotte–Pugh classification, and individual systemic therapies. Current clinical recommendations were reviewed using the National Comprehensive Cancer Network Clinical Practice Guidelines in Oncology. Reference lists of relevant publications were also examined. For this narrative review, study selection was guided by clinical relevance rather than predefined systematic-review eligibility criteria.

## 2. Defining Hepatic Reserve and Hepatic Function in Patients with HCC

Established staging and treatment guidelines for HCC incorporate tumor burden, performance status, and the degree of underlying liver dysfunction into management decisions [1,4,10]. The Barcelona Clinic Liver Cancer (BCLC) staging system, the most widely used framework for HCC management, integrates liver function, tumor burden, and patient performance status to classify patients into five prognostic stages with corresponding treatment recommendations [10]. A limitation of the BCLC system is that underlying liver function is heterogeneous and may be further characterized using established prognostic tools, including the Child–Turcotte–Pugh (CTP) and albumin–bilirubin (ALBI) scores [10,11].

### 2.1. Child–Turcotte–Pugh Classification Considerations in Patients with HCC

The CTP classification was developed as a prognostic tool to assess mortality risk in patients with cirrhosis and portal hypertension undergoing esophageal surgery for variceal bleeding [12]. The CTP classification has subsequently been extended as a tool for assessing underlying liver dysfunction and prognostication of patient mortality across a wide range of liver conditions, including HCC. The CTP classification is widely used to assess underlying liver function and determine patient eligibility for HCC clinical trials and is incorporated into major guidelines [1,4,10].

The CTP classification is derived from five subjective and objective parameters: serum bilirubin, serum albumin, prothrombin time, and the presence and severity of ascites and hepatic encephalopathy [12,13]. Each component is assigned 1–3 points based on severity, generating a total score of 5–15 that stratifies patients into CTP-A (5–6 points), CTP-B (7–9 points; moderate hepatic dysfunction), or CTP-C (10–15 points; advanced hepatic dysfunction) [12,13,14]. Notably, the predicted 1-year survival rates in patients with cirrhosis are approximately 100% for CTP-A, 80% for CTP-B, and 45% for CTP-C [15], which should be taken into consideration when evaluating the competing risks of mortality between liver disease and HCC. These classes have important therapeutic implications: patients with CTP-A disease are potentially eligible for cancer-directed treatment modalities and clinical trials from the standpoint of hepatic function, whereas those with CTP-C disease are typically limited to best supportive care because of poor hepatic reserve [1,4,10]. However, CTP categories are heterogeneous, and in patients with HCC, both tumor burden and underlying cirrhosis may contribute to the score.

Among patients with CTP-A, the CTP classification has limited ability to identify hepatic reserve despite clinically relevant heterogeneity within this population [11,14]. The CTP-B category, encompassing scores from CTP-B7 to CTP-B9, represents a heterogeneous group with different degrees of hepatic impairment, ranging from patients with mildly impaired liver function to those approaching decompensation. In selected patients with CTP-C disease, components of the CTP score may potentially be influenced partly by tumor burden rather than irreversible hepatic dysfunction. Although outcomes are generally poor, emerging real-world evidence supports individualized consideration of systemic therapy in carefully selected CTP-C patients [16].

The CTP classification has important limitations. Two components, ascites and hepatic encephalopathy, are based on subjective clinical assessment, leading to interpretation variability, and their severity may be influenced by treatments such as diuretics, rifaximin, and lactulose [10,14]. In addition, biochemical parameters are equally weighted despite differences in their prognostic significance [14]. The CTP classification does not distinguish ascites related to portal vein tumor involvement, vascular compression, or liver tumor burden from ascites secondary to underlying cirrhosis [14]. The CTP score also omits several clinically relevant prognostic factors, including recurrent variceal bleeding, severe malnutrition, and overall functional status, which are relevant to liver disease-related prognosis and risk assessment for cancer-directed therapy, but not completely captured by albumin and INR values [4,10,14].

### 2.2. The Albumin–Bilirubin (ALBI) Scoring System as an Objective Data-Based Alternative

Recognizing the limitations of the CTP system, the albumin–bilirubin (ALBI) grade was introduced in 2015 as an alternative based exclusively on objective parameters [11]. The ALBI grade is calculated using serum albumin and total bilirubin according to a logarithmic formula, and patients are stratified into ALBI grades 1–3 [11]. A key advantage of the ALBI grade is its ability to discriminate prognostically distinct subgroups within the CTP population [11]. In a landmark international study, ALBI grade identified two groups with clearly different prognoses among CTP-A patients, a distinction not apparent with CTP classification [11]. Subsequent validation studies have reported that ALBI grading provides prognostic information relative to the CTP classification, with better patient distribution across grades and larger survival differences between categories [17]. The combined use of ALBI grading for objective stratification and CTP classification for clinical assessment incorporating ascites and encephalopathy may provide a more comprehensive evaluation of liver function than either system alone [14].

**Figure 2:**
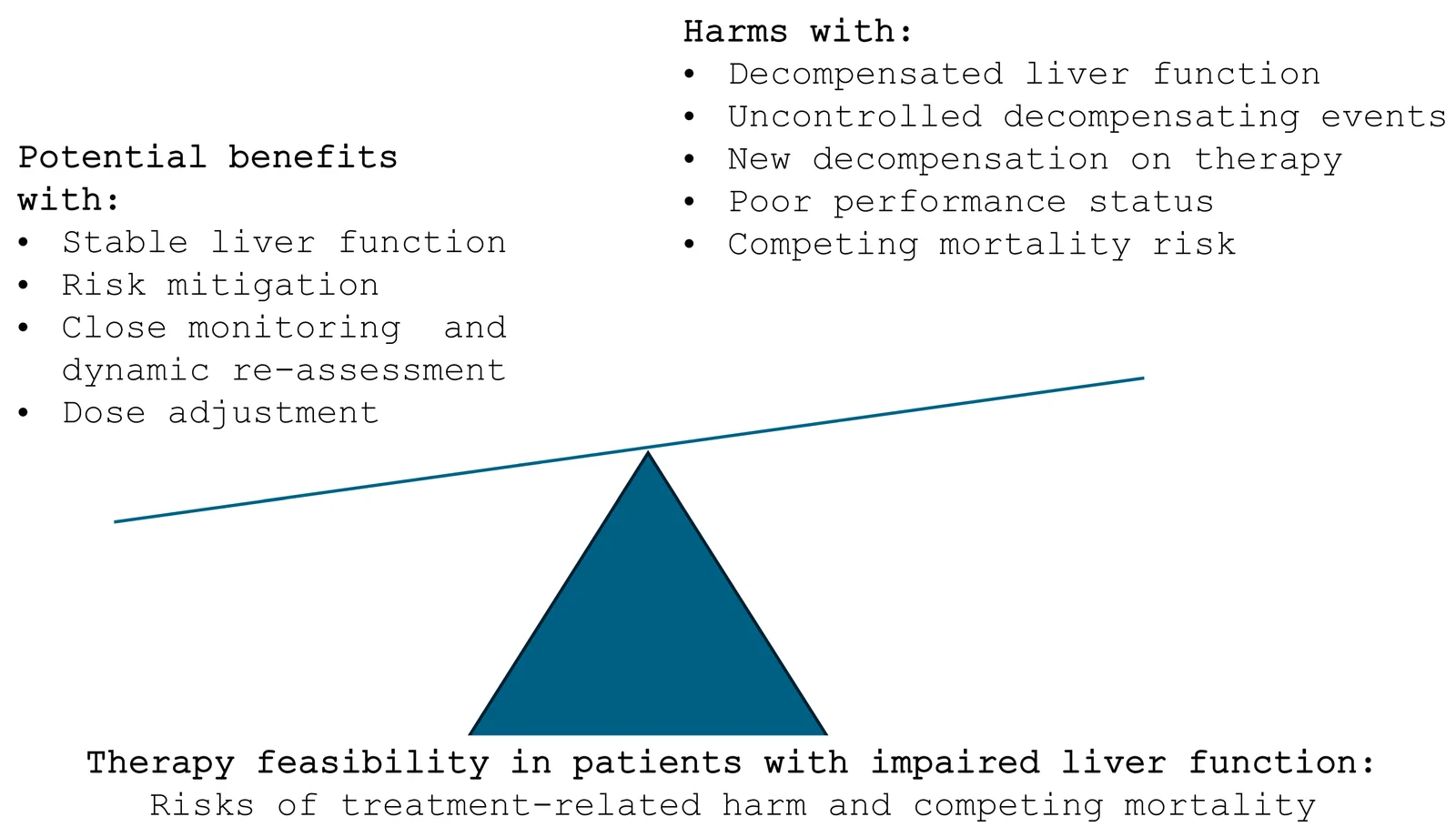
Individualized assessment of systemic therapy feasibility in patients with HCC and impaired liver function.

Beyond ALBI and CTP classification, treatment eligibility requires an individualized assessment considering hepatic reserve and clinical stability against the risks of hepatic decompensation, treatment-related toxicity, and competing mortality from underlying liver disease (Figure 2). Clinical assessment, including evaluation of hepatic encephalopathy, ascites, longitudinal stability of underlying liver disease, and patient functional status, remains central to determining the impact of liver function on prognosis and treatment tolerance, as these clinical parameters may indicate the transition from compensated to decompensated cirrhosis [1,4]. Among these features, ascites is particularly important in HCC, serving as the initial manifestation of decompensation in many patients and as an independent predictor of mortality. Patients classified as CTP-B7 without ascites may remain candidates for systemic therapy [4,18], whereas those with refractory ascites or recurrent encephalopathy face a substantially higher risk of further treatment-related hepatic decompensation [18].

Accordingly, NCCN guidelines incorporate assessment of clinically significant portal hypertension when evaluating hepatic function and treatment eligibility. According to the American Association for the Study of Liver Diseases (AASLD) and European Association for the Study of the Liver (EASL), clinically significant portal hypertension (CSPH) is defined hemodynamically by a hepatic venous pressure gradient (HVPG) ≥10 mmHg [19]. When HVPG measurement is unavailable, noninvasive criteria incorporating liver stiffness measurement (LSM) and platelet count can be used to rule in or rule out CSPH in appropriate populations [19]. Clinical manifestations of decompensation, including ascites, variceal bleeding, and hepatic encephalopathy, as well as gastroesophageal varices, portosystemic collaterals, or hepatofugal flow, provide additional evidence of CSPH [1,4,19]. Taken together, accurate characterization of hepatic reserve requires integration of validated scoring systems with careful clinical assessment (Table 1).

## 3. Immune Checkpoint Inhibitors in Patients with Impaired Liver Function

Immune checkpoint inhibitors (ICIs) present an attractive treatment option for selected patients with impaired liver function, as they undergo minimal hepatic metabolism. They have also demonstrated a favorable safety profile in prospective and retrospective studies. The two prospective trials that evaluated the safety and efficacy of ICIs in patients with HCC and CTP-B liver disease are CheckMate 040 and SIERRA [20,21].

CheckMate 040 is a phase I/II open-label clinical trial of nivolumab for the treatment of advanced HCC. Cohort 5 comprised patients with CTP-B7/B8 disease who were carefully selected based on clinical criteria, including mild ascites, bilirubin < 3 mg/dL, no recent encephalopathy, and no recent paracentesis. Within this cohort, treatment with nivolumab resulted in an objective response rate (ORR) of 12% (per RECIST 1.1 criteria), median progression-free survival (PFS) of 2.7 months, and median overall survival (OS) of 7.6 months. Treatment-related adverse events led to treatment discontinuation in a small percentage of patients (4%). Efficacy of nivolumab was similar in sorafenib-naïve and previously sorafenib-treated patients [20].

The SIERRA trial is a phase 3b study assessing the safety and efficacy of the STRIDE regimen (single-dose tremelimumab with durvalumab every 4 weeks) specifically in patients with unresectable advanced HCC who had not received prior systemic therapy and who had poor prognostic factors, including CTP-B7/B8, ECOG performance status 2, or main trunk portal vein thrombosis. Early safety analysis of SIERRA showed that, among patients with CTP-B7/B8 disease, the incidence of possibly related to study treatment adverse events (PRAEs) was 48.6%, with 2.9% being serious PRAEs. No PRAEs led to death in patients with CTP-B7/B8 disease. The most common adverse events were diarrhea, pruritus, and AST elevation [21].

Additional studies that explored outcomes in patients with HCC, CTP-A disease, and ALBI grade 2 or greater liver dysfunction in post hoc analyses include the HIMALAYA and IMbrave150 randomized clinical trials. HIMALAYA is a randomized, open-label phase III trial of the STRIDE regimen compared with durvalumab every 4 weeks and sorafenib in patients with unresectable HCC who had not previously received systemic treatment [7]. Subgroup analysis of trial patients stratified by baseline liver function showed higher OS rates across ALBI grades in patients receiving STRIDE. In patients with ALBI grade 1, the 5-year OS rate was 24.3% with STRIDE compared with 13.6% with sorafenib, while in patients with ALBI grade 2/3, the 5-year OS rate was 13.7% with STRIDE compared with 4.7% with sorafenib, maintaining a survival benefit for STRIDE relative to sorafenib [22]. Of note, in patients with ALBI grade 2/3, the 5-year OS rate with durvalumab alone was comparable to that with STRIDE combination immunotherapy, at 13.2%, whereas in ALBI grade 1 patients, the 5-year OS rate was lower at 15.5% with durvalumab alone compared with 24.3% with STRIDE [22]. Treatment-related serious adverse events were slightly higher in patients receiving STRIDE (ALBI grade 1, 20.4%; ALBI grade 2/3, 14.0%) compared with sorafenib (ALBI grade 1, 8.1%, and ALBI grade 2/3, 11.9%) and durvalumab alone (ALBI grade 1, 7.1%, and ALBI grade 2/3, 10.0%) [22].

The IMbrave150 study, a phase III open-label multicenter trial, was the first phase III study to demonstrate the superiority of an immunotherapy-based combination over sorafenib in advanced HCC. It investigated the efficacy of atezolizumab plus bevacizumab compared with sorafenib in patients with advanced HCC who had not previously received systemic therapy and demonstrated improved OS and PFS in patients receiving atezolizumab plus bevacizumab [6]. Notably, patients considered for atezolizumab plus bevacizumab require careful pretreatment evaluation for esophageal varices, given the risk of bleeding with bevacizumab treatment [6]. The study enrolled patients with CTP-A disease. A post hoc analysis assessing the impact of ALBI grade showed that, among ALBI grade 1 patients, those receiving atezolizumab plus bevacizumab had markedly improved OS, with median OS not estimable after up to 24 months of follow-up, compared with 15.4 months (95% CI, 11.7–20.8) in the sorafenib group. However, among ALBI grade 2 patients, the atezolizumab–bevacizumab and sorafenib arms had similar median OS, at 11.7 months (95% CI, 9.1–16.1) versus 12.2 months (95% CI, 7.2–16.1), respectively [23].

Notably, ALBI grade 1 patients had a longer duration of treatment, receiving a median of 10.4 months of atezolizumab compared with 6.1 months for ALBI grade 2 patients, and 9.6 months of bevacizumab compared with 4.9 months for ALBI grade 2 patients, although the median duration of sorafenib treatment was similar (2.9 months for ALBI grade 1 patients and 2.8 months for ALBI grade 2 patients), demonstrating shorter treatment exposure in patients with ALBI grade 2 compared with ALBI grade 1. Rates of TRAEs were similar in the atezolizumab plus bevacizumab groups, with 48% of ALBI grade 1 patients and 38% of ALBI grade 2 patients experiencing grade 3–4 TRAEs, the most common of which were hypertension and AST elevation [23].

A multicenter retrospective review of patients with advanced HCC treated with atezolizumab plus bevacizumab according to the IMbrave150 regimen found no difference in the frequency of TRAEs between CTP-A and CTP-B cohorts (grade >3 TRAEs secondary to bevacizumab in 16% and 15% of patients, respectively; grade >3 TRAEs secondary to atezolizumab in 15% and 4% of patients, respectively). ORRs were also similar, at 26% in CTP-A patients and 21% in CTP-B patients. However, median OS was 16.8 months (95% CI, 14.1–23.9) in CTP-A patients and 6.7 months (95% CI, 4.3–15.6) in CTP-B patients [24].

The randomized CheckMate 9DW study of nivolumab plus ipilimumab versus sorafenib or lenvatinib in patients with advanced HCC enrolled patients with CTP-A disease [25]. Grade 3–4 TRAEs occurred in 41% of patients receiving nivolumab plus ipilimumab compared with 42% receiving lenvatinib or sorafenib, and treatment-related deaths occurred in 12 and 3 patients, respectively [25]. Data on nivolumab plus ipilimumab in patients with impaired liver function are limited; therefore, safety data in this population are needed before consideration of its use in patients with liver dysfunction.

In summary, based on the limited available data, selected patients with impaired liver function with CTP-A and ALBI grade 2/3 or CTP-B disease may be considered for treatment with ICIs under close supervision by a multidisciplinary team. Among ICI-only regimens, the STRIDE regimen (tremelimumab plus durvalumab), durvalumab, and nivolumab have prospective safety and efficacy data in the populations described above [20,21]. Atezolizumab plus bevacizumab requires separate consideration because of the anti-VEGF component and associated bleeding risk; evidence for its use in CTP-B disease is primarily derived from retrospective studies [24]. Noninvasive objective assessment using liver stiffness measurement (LSM) and clinical evaluation for clinically significant portal hypertension, along with preventive measures (endoscopy with variceal banding or consideration of β-blockers), can be conducted to assess risks associated with underlying liver disease and treatment-related adverse events [1,4,19]. Selected patients with impaired liver function may tolerate systemic therapy, but treatment-related adverse events and hepatic decompensation require close monitoring.

## 4. Tyrosine Kinase Inhibitors in Patients with Impaired Liver Function

Unlike immune checkpoint inhibitors, tyrosine kinase inhibitors (TKIs) undergo hepatic CYP450 metabolism, making tolerability dependent in part on hepatic function. TKIs demonstrate activity in selected patients with impaired liver function; however, worsening liver impairment is associated with shorter TKI treatment duration, progressive hepatic decompensation, and decreased survival. While TKIs were the first agents approved for advanced HCC, they are now more commonly used as second- or later-line treatment after progression on immunotherapy or in patients who are not candidates for immunotherapy.

### 4.1. Sorafenib and Lenvatinib

Sorafenib was the first TKI approved for use in HCC in 2007. The GIDEON study was a large prospective observational study that compared outcomes among patients with different CTP classes treated with sorafenib. This study demonstrated a correlation between worsening CTP class and shorter median OS, with a median OS of 13.6 months in CTP-A patients, 5.2 months in CTP-B patients, and 2.6 months in CTP-C patients [9]. Moreover, within CTP-B, there was a trend toward worse median OS in CTP-B9 compared with CTP-B8 and CTP-B7 patients, highlighting the heterogeneity of the CTP-B category [9]. Serious adverse events were more frequent in CTP-B than in CTP-A patients (60% vs. 36%, respectively) [9]. The most notable adverse effects of sorafenib included diarrhea and hand–foot syndrome, which limited tolerability [9]. These findings highlight diminishing treatment benefit in the setting of worsening hepatic reserve and increasing toxicity. In patients with CTP-C disease and advanced HCC, supportive and palliative care, with a focus on quality of life and symptom control, is a reasonable step in management, while selected patients with CTP-B disease could be considered for treatment with close multidisciplinary monitoring.

Lenvatinib was the next TKI approved for HCC, almost a decade after sorafenib. It was approved based on the REFLECT trial, a phase III open-label noninferiority trial evaluating the efficacy of lenvatinib compared with sorafenib. The trial demonstrated a median OS of 13.6 months (95% CI, 12.1–14.9) in the lenvatinib arm compared with 12.3 months (95% CI, 10.4–13.9) in the sorafenib arm in patients with CTP-A liver function [26]. In a retrospective analysis of REFLECT patients comparing those who deteriorated from CTP-A to CTP-B while on treatment with those who remained CTP-A, CTP-B patients had a lower ORR than CTP-A patients (28.3% and 42.9%, respectively). Median OS was also lower in CTP-B patients than in CTP-A patients, at 6.8 months (95% CI, 2.6–10.3) compared with 13.3 months (95% CI, 11.6–16.1), respectively [27]. These findings suggest that preserved liver function may be associated with more favorable outcomes during lenvatinib treatment, potentially reflecting both improved treatment tolerability and the overall better prognosis associated with preserved hepatic reserve.

The LAUNCH prospective study examined the safety and efficacy of lenvatinib in a real-world setting and therefore included CTP-B patients. It demonstrated a higher incidence of grade ≥ 3 adverse events (100% compared with 80.9% in CTP-A patients), and higher discontinuation rates (33.3% in CTP-B patients compared with 17.0% in CTP-A patients). Median PFS was 6.4 months in CTP-A patients and 2.5 months in CTP-B patients, while median OS was 19.7 months in CTP-A patients and 4.1 months in CTP-B patients, respectively. Of note, lenvatinib plasma concentrations were higher in CTP-B patients compared with patients with CTP-A disease [28], suggesting the importance of considering dose reduction in lenvatinib in patients with impaired liver function (CTP-B) and closely monitoring for the development of drug-related toxicities.

### 4.2. Cabozantinib and Regorafenib

Cabozantinib and regorafenib were FDA-approved for patients who had previously been treated with sorafenib based on the following trials. The CELESTIAL study was a phase III randomized, double-blind, controlled trial of cabozantinib versus placebo in patients with CTP-A and advanced HCC who had previously been treated with sorafenib. The study demonstrated improved median OS of 10.2 months in patients who received cabozantinib compared with 8.0 months in patients receiving placebo (HR, 0.76; 95% CI, 0.63–0.92) [29]. In a post hoc exploratory analysis evaluating outcomes among patients who deteriorated from CTP-A to CTP-B by week 8, survival was longer among those receiving cabozantinib than among those receiving placebo, with an OS of 8.5 months compared with 3.8 months in the placebo group (HR, 0.32; 95% CI, 0.18–0.58) and a PFS of 3.7 months compared with 1.9 months, respectively (HR, 0.44; 95% CI, 0.25–0.76) [30].

REFINE was a prospective multicenter study evaluating the safety and efficacy of regorafenib in a broad population of patients with unresectable HCC who had received prior systemic therapy in real-world clinical practice. Median OS was 15.5 months (95% CI, 13.9–16.3) in CTP-A patients and 6.3 months (95% CI, 4.9–7.8) in CTP-B patients. Overall, 54% of patients experienced a grade ≥3 adverse event, and 27% experienced a grade ≥3 TRAE. The most common TRAEs were hand–foot skin reaction, diarrhea, and fatigue. Treatment discontinuation due to adverse events occurred in 31% of patients, numerically higher than the 25% rate in the RESORCE trial, which did not include CTP-B patients [3,31]. Full-dose therapy maintenance was limited, with only 54% of patients who started on the standard regorafenib dose of 160 mg daily remaining on that dose throughout treatment. Patients receiving regorafenib as second-line treatment had longer median OS than those receiving it as third-line or later therapy (13.9 months, 95% CI, 12.2–15.3 after first-line sorafenib; 17.4 months, 95% CI, 6.8–21.6 after first-line non-sorafenib therapy; and 9.7 months, 95% CI, 7.9–13.9 as later-line therapy) [31].

A meta-analysis of eight studies involving 809 patients reported outcomes with regorafenib; however, most participants had CTP-A liver function, and the small number with CTP-B disease precluded subgroup-specific efficacy assessment [32].

## 5. Ramucirumab in Patients with Impaired Liver Function

Although the VEGFR-2 inhibitor ramucirumab demonstrated modest improvement in OS compared with placebo in patients previously treated with sorafenib and with alpha-fetoprotein values ≥400 ng/mL, trials to date have included only patients with CTP-A liver disease; therefore, there is insufficient evidence to recommend routine use in patients with more impaired liver function [33].

## 6. Overall Considerations for Systemic Therapy in Patients with Impaired Liver Function

Overall, the evidence for systemic therapy, including TKIs and immune checkpoint inhibitors, in patients with impaired liver function suggests that treatment is feasible in selected patients with CTP-B disease, with consideration of TKI dose reduction and close monitoring for drug-related toxicities and liver function (Table 2).

However, compared with immunotherapy, hepatic reserve may have a greater impact on TKI tolerability, including treatment duration, dose intensity, and reported outcomes. For patients with CTP-A disease and ALBI grade 2/3, available subgroup analyses suggest a maintained survival benefit with durvalumab or durvalumab plus tremelimumab relative to sorafenib, whereas median OS was similar with atezolizumab plus bevacizumab vs. sorafenib in the ALBI grade 2 subgroup [22,23]. Given the limited and heterogeneous evidence, these considerations should not be interpreted as a validated treatment algorithm or formal clinical guideline. Treatment selection should remain individualized according to hepatic reserve, clinical stability, tumor characteristics, and patient preferences.

Overall survival estimates derived from randomized trials, prospective cohorts, retrospective series, and registry studies should not be directly compared because of differences in study design, patient selection, baseline tumor burden, performance status, hepatic reserve, treatment line, and subsequent therapies. In addition, the available studies cannot reliably separate the prognostic effect of impaired liver function from the efficacy of systemic therapy. Consequently, shorter overall survival may reflect competing liver-related mortality, tumor burden, or reduced treatment exposure rather than reduced antitumor activity alone. Overall survival estimates across studies should therefore not be directly compared or interpreted as establishing comparative treatment efficacy.

Studies enrolling patients with baseline CTP-B liver function should be distinguished from post hoc analyses of patients who entered trials with CTP-A liver function and deteriorated to CTP-B during treatment. CheckMate 040, SIERRA, GIDEON, LAUNCH, REFINE, and retrospective atezolizumab–bevacizumab cohorts included patients with baseline CTP-B liver function. In contrast, the REFLECT and CELESTIAL analyses evaluated patients who deteriorated from CTP-A to B during treatment. The latter populations had sufficient hepatic reserve to meet the original trial eligibility criteria and should not be considered equivalent to patients presenting with baseline CTP-B disease.

Within the CTP-B population, the available prospective evidence is most applicable to carefully selected patients with B7 or B8 liver function, stable laboratory values, no or controlled ascites, and no recent encephalopathy. Evidence becomes increasingly limited with worsening hepatic reserve and is particularly sparse for B9 disease. Refractory ascites, recurrent encephalopathy, recent variceal bleeding, or progressive hepatic deterioration indicate a higher risk of further decompensation during treatment. Evidence supporting systemic therapy in CTP-C disease remains very limited, and supportive and palliative care focused on symptoms and quality of life is appropriate for most patients.

The evidence in patients with CTP-B liver function is derived from retrospective cohorts, single-arm studies, or post hoc analyses of trials that predominantly enrolled patients with CTP-A disease. These studies are vulnerable to selection bias and residual confounding and should not be interpreted as establishing comparative efficacy or safety in the broader population with impaired hepatic reserve. Patients with CTP scores driven partly by potentially reversible tumor-related factors, such as extensive hepatic involvement or vascular obstruction, may differ clinically and prognostically from patients with advanced irreversible cirrhosis. Because available studies do not consistently distinguish these populations, interpreting their outcomes without accounting for these differences may confound the relationships among hepatic reserve, treatment tolerance, and survival.

Clinically important outcomes, including time to first or worsening hepatic decompensation, new or worsening ascites or encephalopathy, variceal bleeding, deterioration in CTP class or albumin–bilirubin grade, functional decline, hospitalizations, treatment discontinuation and quality of life, were inconsistently reported. This limits assessment of overall clinical benefit because maintaining treatment without precipitating hepatic decompensation may be as clinically meaningful as tumor response or overall survival. Future prospective studies should incorporate these outcomes as predefined endpoints. Available studies do not consistently report outcomes according to liver disease etiology, frailty, or sarcopenia, limiting stratification by these clinically relevant factors.

Preclinical research continues to identify potential therapeutic approaches in hepatocellular carcinoma, including modulation of the tumor immune microenvironment through the glucocorticoid receptor and targeting extracellular matrix proteins such as lumican [34,35]. However, the clinical relevance of these approaches, particularly in patients with impaired liver function, remains to be established.

## 7. Conclusions

Patients with impaired liver function are a heterogeneous group regarding liver impairment severity and manifestation, and assessment of liver function in patients with HCC extends beyond standard stratification systems, including the Child–Turcotte–Pugh (CTP) classification and albumin–bilirubin (ALBI) grade, and requires careful, ongoing clinical assessment to determine the risk–benefit balance of systemic therapy. Despite advances in modern therapies for advanced HCC, underlying liver disease and impaired liver function remain competing risks for mortality.

In this review, we highlighted the available evidence and its limitations for the treatment of HCC in patients with impaired liver function. Future progress depends on prospective trials that include CTP-B cohorts and incorporate survival and time to hepatic decompensation as clinical trial endpoints. Ultimately, as systemic therapies become more effective, the best systemic therapy for patients with HCC and impaired liver function is one that the patient can safely remain on long enough to derive antitumor benefit. This requires informed, shared decision-making. For patients with impaired liver function who can safely tolerate systemic therapy, nuanced patient evaluation and close monitoring for hepatic decompensation are as essential as monitoring tumor response.

## Figures and Tables

**Table 1:** Assessment of Hepatic Reserve before Systemic Therapy.

Variable	Clinical Findings	Assessment	Significance
Child–Turcotte–Pugh (CTP)	Albumin, bilirubin, INR, ascites, encephalopathy	CTP-A vs. CTP-B7 vs. CTP-B8/9 vs. CTP-C	CTP-B heterogeneous, with differences in prognosis and treatment tolerance
ALBI	Albumin and bilirubin	ALBI grade 1 vs. 2 vs. 3	Objective assessment; discrimination of hepatic reserve within CTP-A
Ascites	None, controlled, refractory	Controlled vs. uncontrolled	Marker of decompensation and reduced treatment tolerance
Bilirubin	Objective value and longitudinal trend	Stable vs. improving vs. increasing	Trajectory of hepatic reserve and tumor burden
Esophageal varices	Prior bleeding history and EGD findings	Low vs. high GI bleeding risk	Eligibility evaluation for anti-angiogenesis bevacizumab
Hepatic encephalopathy	History and current status	Controlled vs. active	Hepatic reserve marker, may affect functional status
Tumor burden	Tumor in vein and extent of liver involvement	Tumor-related vs. cirrhosis-related dysfunction	May improve with tumor response; may increase bleeding risk
Liver disease stability	Decompensation history, hospitalizations, AST/ALT/bilirubin trends	Stable vs. deteriorating	Dynamic prognosis of hepatic reserve, treatment tolerance, need for dose adjustment
Performance status	ECOG	ECOG 0–1 vs. 2 vs. 3–4	Treatment tolerance; may be influenced by liver disease

Abbreviations: ALBI, albumin–bilirubin grade; ALT, alanine aminotransferase; AST, aspartate aminotransferase; CTP, Child–Turcotte–Pugh; ECOG, Eastern Cooperative Oncology Group; EGD, esophagogastroduodenoscopy; GI, gastrointestinal; INR, international normalized ratio.

**Table 2:** Summary of Evidence for Systemic Therapy in HCC with Impaired Liver Function.

Agent	Evidence Type	Impaired Liver Function Included	Survival Outcomes	Key Considerations
Sorafenib	Prospective Observational study: GIDEON	CTP-A/B/C	Median OS: CTP-A, 13.6 mo; CTP-B, 5.2 mo (CTP-B7, 6.2 mo; CTP-B8, 4.8 mo; CTP-B9, 3.7 mo); CTP-C, 2.6 mo	Shorter OS with worsening CTP class
Durvalumab + Tremelimumab (STRIDE)	Prospective: Phase 3b trial SIERRA; Post hoc analysis, prospective trial: HIMALAYA * ALBI	SIERRA: CTP-B7/B8; HIMALAYA: CTP-A (ALBI grade 1 vs. 2/3)	HIMALAYA 5-year OS: ALBI grade 1, 24.3% with STRIDE vs. 13.6% with sorafenib; ALBI grade 2/3, 13.7% vs. 4.7%	Manageable safety in selected CTP-B7/B8 patients; HIMALAYA supports efficacy across ALBI subgroups.
Nivolumab	Prospective trial: CheckMate 040, Cohort 5	CTP-B7/8	Median OS: 7.6 mo	Selected CTP-B population
Atezolizumab+ Bevacizumab	Post hoc analysis, prospective trial: IMbrave150 * ALBI analysis Retrospective: CTP-B studies	IMbrave150: CTP-A (ALBI grade 1 vs. 2); retrospective: CTP-A/B	IMbrave150 ALBI grade 2: median OS, 11.7 vs. 12.2 mo with sorafenib; retrospective: 16.8 mo CTP-A vs. 6.7 mo CTP-B	Bleeding risk; pretreatment variceal evaluation, consider variceal bleeding prevention strategies
Lenvatinib	Post hoc analysis, prospective trial: REFLECT * Prospective real-world: LAUNCH	REFLECT: deteriorating CTP-A → CTP-B LAUNCH: CTP-A/B	REFLECT: median OS, 6.8 mo CTP-B vs. 13.3 mo CTP-A; LAUNCH: 4.1 mo CTP-B vs. 19.7 mo CTP-A	Higher drug exposure in CTP-B; dose-intensity concerns
Regorafenib	Prospective observational study: REFINE	CTP-A/B	Median OS: 15.5 mo CTP-A vs. 6.3 mo CTP-B	Limited maintenance of full-dose therapy
Cabozantinib	Post hoc analysis, prospective trial: CELESTIAL *	Deterioration from CTP-A → CTP-B by week 8	Median OS 8.5 mo cabozantinib vs. 3.8 mo placebo	Does not represent patients with baseline CTP-B

* Randomized trials enrolled patients with CTP-A liver function; impaired liver function data shown are derived from post hoc analyses. Abbreviations: ALBI, albumin–bilirubin grade; CTP, Child–Turcotte–Pugh; OS, overall survival; mo, months. Evidence type reflects the design of the cited study and does not represent a formal evidence-grade assessment. Outcomes are stratified by individual CTP-B score when reported in the original study.

## Data Availability

No new data were created or analyzed in this study. Data sharing is not applicable to this article.
